# 2,2′-[(*E*,*E*)-1,1′-(2,2-Dimethyl­propane-1,3-diyldinitrilo)diethyl­idyne]diphenol

**DOI:** 10.1107/S1600536809022855

**Published:** 2009-06-20

**Authors:** Morteza Montazerozohori, Mohammad Hossein Habibi, Ahmad Hojjati, Reza Mokhtari, Yuki Yamane, Takayoshi Suzuki

**Affiliations:** a Department of Chemistry, Yasouj University, Yasouj, 75914-353, Iran; b Catalysis Division, Department of Chemistry, University of Isfahan, Isfahan 81746-73441, Iran; c Department of Chemistry, Faculty of Science, Okayama University, Tsushima-naka 3-1-1, Okayama 700-8530, Japan

## Abstract

The title Schiff base, C_21_H_26_N_2_O_2_, contains two intra­molecular O—H⋯N hydrogen bonds between the hydroxyl groups and the nearest imine N atoms, each leading to a six-membered ring. Weak C—H⋯O hydrogen bonds result in a ladder network running along the *a* axis. In addition, inter­molecular C—H⋯π inter­actions serve to stabilize the extended structure.

## Related literature

For the biological activity of Schiff bases, see: Singh & Dash (1988 ▶); More *et al.* (2001 ▶); Baseer *et al.* (2000 ▶); El-Masry *et al.* (2000 ▶); Kabeer *et al.* (2001 ▶); Kuzmin *et al.* (2000 ▶); Desai *et al.* (2001 ▶). For metal complexes of Schiff bases, see: Habibi *et al.* (2007*a* ▶)*. *For related structures, see: Barati *et al.* (2009 ▶); Habibi *et al.* (2007*b* ▶,*c* ▶).
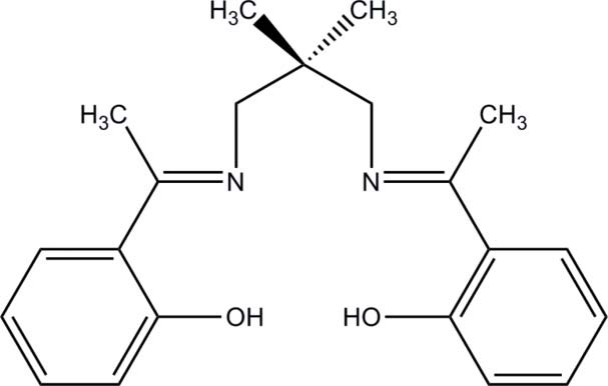


## Experimental

### 

#### Crystal data

C_21_H_26_N_2_O_2_
*M* *_r_* = 338.44
Triclinic, 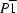

*a* = 7.7847 (9) Å
*b* = 9.1857 (12) Å
*c* = 13.3801 (14) Å
α = 79.547 (4)°
β = 77.508 (3)°
γ = 85.537 (4)°
*V* = 917.89 (18) Å^3^
*Z* = 2
Mo *K*α radiation
μ = 0.08 mm^−1^
*T* = 193 K
0.40 × 0.30 × 0.10 mm

#### Data collection

Rigaku R-AXIS RAPID diffractometer
Absorption correction: multi-scan (*ABSCOR*; Higashi, 1995 ▶) *T*_min_ = 0.969, *T*_max_ = 0.992
9076 measured reflections
4143 independent reflections
3022 reflections with *I* > 2σ(*I*)
*R*_int_ = 0.023

#### Refinement

*R*[*F* ^2^ > 2σ(*F* ^2^)] = 0.043
*wR*(*F* ^2^) = 0.135
*S* = 1.05
4143 reflections
331 parameters
All H-atom parameters refined
Δρ_max_ = 0.28 e Å^−3^
Δρ_min_ = −0.17 e Å^−3^

### 

Data collection: *PROCESS-AUTO* (Rigaku, 1998 ▶); cell refinement: *PROCESS-AUTO*; data reduction: *CrystalStructure* (Rigaku/MSC, 2004 ▶); program(s) used to solve structure: *SIR2004* (Burla *et al.*, 2005 ▶); program(s) used to refine structure: *SHELXL97* (Sheldrick, 2008 ▶); molecular graphics: *ORTEP-3 for Windows* (Farrugia, 1997 ▶); software used to prepare material for publication: *SHELXL97*.

## Supplementary Material

Crystal structure: contains datablocks I, global. DOI: 10.1107/S1600536809022855/fj2213sup1.cif

Structure factors: contains datablocks I. DOI: 10.1107/S1600536809022855/fj2213Isup2.hkl

Additional supplementary materials:  crystallographic information; 3D view; checkCIF report

## Figures and Tables

**Table 1:** Hydrogen-bond geometry (Å, °)

*D*—H⋯*A*	*D*—H	H⋯*A*	*D*⋯*A*	*D*—H⋯*A*
O1—H01⋯N1	1.01 (2)	1.56 (2)	2.517 (1)	156 (2)
O2—H02⋯N2	1.02 (2)	1.59 (2)	2.526 (1)	151 (2)
C19—H19⋯O1^i^	0.99 (2)	2.54 (2)	3.488 (2)	174 (1)
C3—H3⋯O2^ii^	0.97 (2)	2.61 (2)	3.291 (2)	128 (1)

Symmetry codes: (i) 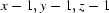; (ii) 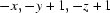.

**Fig. 1.**
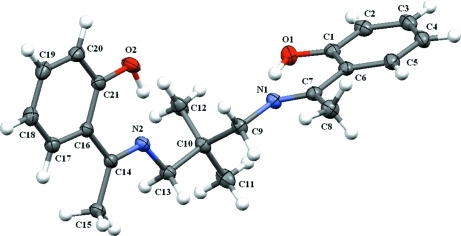
A view of the title compound, showing the atom-numbering scheme. Displacement ellipsoids are drawn at the 30% probability level and H atoms are shown as small spheres of arbitrary radii. The intramolecular hydrogen bond is depicted by a dashed line.

**Fig. 2.**
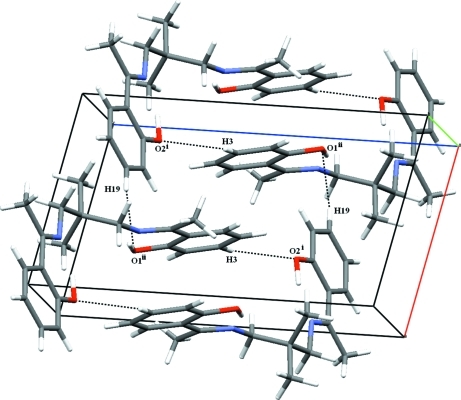
The C–H···O weak intermolecular hydrogen bond polymeric ladder like chains of (I). [symmetry code: (i) 1 - x, 1 - y, 1 - z, symmetry code: (ii) 1 - x, 1 + y, 1 - z.]

**Fig. 3.**
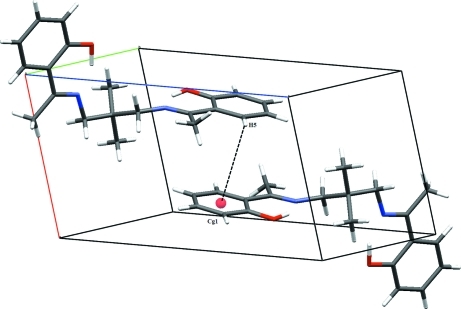
The C–H···π interactions in the crystal structure of (I) involving the ring through atoms C1—C6 (centroid Cg1). (Symmetry code for the interaction: -x, 1 + y, 1 - z)

## supplementary crystallographic information

### Comment

Schiff bases rank among the most versatile synthetic organic intermediates. They are reported to show avariety of biological activities including antifungal (Singh & Dash, 1988; More *et al.*, 2001), antibacterial (Baseer *et al.*, 2000; El-Masry *et al.*, 2000; Kabeer *et al.*, 2001) and anticancer (Kuzmin *et al.*, 2000; Desai *et al.*, 2001) among others.

Schiff bases and their metal complexes play a key role in understanding the coordination chemistry of transition-metal ions (Habibi *et al.*, 2007*a*).

Relatively few crystal structures have been reported for tetradentate Schiff base ligands and complexes (Habibi *et al.*, 2007*b*,c; Barati *et al.*, 2009).

salen type Schiff bases have enol–keto tautomerism. Depending on the type of tautomer, two types of intramolecular hydrogen bond involving a photochemically or thermochemically induced proton transfer are possible, namely O···H–N in keto (NH) and O–H···N in enol (OH) tautomers. Although the proton transfer reaction is seemingly straight forward, it causes a change in the π electronic system and induces large-scale in-plane and out-of-plane skeletal deformations.

The molecular structure of the ligand is represented in figure 1. The bond lengths and angles are in eligible range. Furthermore, the shortest N–O distances [average 2.521 (3) Å] are indicative of intramolecular hydrogen bonding, as indicated by the dotted lines in the figure 1. The N1–C9 [1.4622 (15) Å] and N1–C7 [1.2833 (15) Å] bonds show typical values of C–N and C=N bonds, respectively.

The C1–C6 benzene ring is nearly perpendicular to C16–C21 benzene ring and makes the dihedral angle of 86.53 (5)°. The C7–N1–C9–C10 and C14–N2–C13–C10 torsion angles are -158.41 (11) and 178.35 (10)°, respectively, and the C6–C7–N1–C9 and C16–C14–N2–C13 torsion angles are177.71 (10) and -179.70 (10)°, respectively.

There are two strong intramolecular hydrogen bonds [O1–H1···N1 and O2–H2···N2], (Table1 and Fig. 1), and each of them serves to stabilize the geometry of the molecule.

The molecules of (I) are packed into one-dimensional polymeric ladder like arrangements generated by translation along *a* axis of the unit cell with the aid of weak C3–H3···O2^i^ and C19–H19···O1^ii ^hydrogen bonds [symmetry code: (i) 1 - *x*, 1 - *y*, 1 - *z*, symmetry code: (ii) 1 - *x*, 1 + *y*, 1 - *z*] (Fig. 2). A noteworthy intermolecular C–H···π interaction (Fig. 3) involving the ring through atoms C1—C6 (centroid *Cg*1), *Cg*1···*Cg*1[symmetry code: -*x*, 1 + *y*, 1 - *z*], supplies a principal contribution to the molecular packing.

### Experimental

To 1 mmol of 2,2-dimethyl-1,3-propanediamine in 15 ml e thanol, 2 mmol of 2-hydroxyacetophenone in 15 ml e thanol was added. The reaction mixture was refluxed for 4 h. Then the mixture was kept in refrigerator overnight and then filtered to give the product as yellow crystals suitable for X-ray single-crystal in 92% yields. The product was characterized by physical and spectral data. Elemental analysis, %C_21_H_26_N_2_O_2_: calculated: C, 74.52; H, 7.74; N, 8.28; O, 9.45; found: C, 74.55; H, 7.68; N, 8.27. IR (KBr, cm^-1^): 3442 (m, –OH), 3042 (m,CH-aromatic), 2982 (m, CH-aliphatic), 2962 (m, CH-aliphatic), 2903 (m,CH-aliphatic), 2833 (m, CH-iminic), 1614(*versus*, –C=N(*asym*)), 1579 (s, –C=N(*sym*)),1509 (*s*), 1449 (*s*), 1304(*s*), 1259 (*m*), 1238 (*m*), 1155 (*s*), 1061 (s, C—O), 936 (*m*),839 (*s*), 759 (*versus*), 634 (*m*), 581(*m*), 523 (*m*), 503 (*m*), 411(w). UV [EtOH, λnm(ε)]: 390 (0.70× 10^6^), 321 (0.64× 10^6^), 276 (shoulder, 1.37× 10^6^), 254 (1.94× 10^6^).

### Refinement

All H atoms were located in the subsequent difference Fourier maps and they were refined with isotopic thermal parameters.

### Crystal data

**Table:**

C_21_H_26_N_2_O_2_	*Z* = 2
*M**_r_* = 338.44	*F*(000) = 364
Triclinic, *P*1	*D*_x_ = 1.225 Mg m^−^^3^
*a* = 7.7847 (9) Å	Mo *K*α radiation, λ = 0.71075 Å
*b* = 9.1857 (12) Å	Cell parameters from 6952 reflections
*c* = 13.3801 (14) Å	θ = 3.2–27.5°
α = 79.547 (4)°	µ = 0.08 mm^−^^1^
β = 77.508 (3)°	*T* = 193 K
γ = 85.537 (4)°	Block, yellow
*V* = 917.89 (18) Å^3^	0.40 × 0.30 × 0.10 mm

### Data collection

**Table:**

Rigaku R-AXIS RAPID diffractometer	4143 independent reflections
Radiation source: fine-focus sealed tube	3022 reflections with *I* > 2σ(*I*)
graphite	*R*_int_ = 0.023
Detector resolution: 10.00 pixels mm^-1^	θ_max_ = 27.5°, θ_min_ = 3.2°
ω scans	*h* = −9→10
Absorption correction: multi-scan (*ABSCOR*; Higashi, 1995)	*k* = −11→11
*T*_min_ = 0.969, *T*_max_ = 0.992	*l* = −17→17
9076 measured reflections	

### Refinement

**Table:**

Refinement on *F*^2^	Primary atom site location: structure-invariant direct methods
Least-squares matrix: full	Secondary atom site location: difference Fourier map
*R*[*F*^2^ > 2σ(*F*^2^)] = 0.043	Hydrogen site location: difference Fourier map
*wR*(*F*^2^) = 0.135	All H-atom parameters refined
*S* = 1.05	*w* = 1/[σ^2^(*F*_o_^2^) + (0.0886*P*)^2^] where *P* = (*F*_o_^2^ + 2*F*_c_^2^)/3
4143 reflections	(Δ/σ)_max_ < 0.001
331 parameters	Δρ_max_ = 0.28 e Å^−^^3^
0 restraints	Δρ_min_ = −0.16 e Å^−^^3^

### Special details

**Table:**

Geometry. All e.s.d.'s (except the e.s.d. in the dihedral angle between two l.s. planes) are estimated using the full covariance matrix. The cell e.s.d.'s are taken into account individually in the estimation of e.s.d.'s in distances, angles and torsion angles; correlations between e.s.d.'s in cell parameters are only used when they are defined by crystal symmetry. An approximate (isotropic) treatment of cell e.s.d.'s is used for estimating e.s.d.'s involving l.s. planes.
Refinement. Refinement of *F*^2^ against ALL reflections. The weighted *R*-factor *wR* and goodness of fit *S* are based on *F*^2^, conventional *R*-factors *R* are based on *F*, with *F* set to zero for negative *F*^2^. The threshold expression of *F*^2^ > σ(*F*^2^) is used only for calculating *R*-factors(gt) *etc*. and is not relevant to the choice of reflections for refinement. *R*-factors based on *F*^2^ are statistically about twice as large as those based on *F*, and *R*- factors based on ALL data will be even larger.

### Fractional atomic coordinates and isotropic or equivalent isotropic displacement parameters (Å^2^)

**Table:**

	*x*	*y*	*z*	*U*_iso_*/*U*_eq_	
O1	0.15478 (13)	0.65393 (11)	0.31309 (7)	0.0451 (2)	
O2	−0.20734 (13)	0.07117 (12)	0.24962 (8)	0.0540 (3)	
N1	0.21630 (13)	0.37811 (11)	0.34121 (8)	0.0364 (2)	
N2	0.11114 (13)	0.06677 (11)	0.15578 (8)	0.0362 (2)	
C1	0.19076 (15)	0.65208 (14)	0.40716 (9)	0.0359 (3)	
C2	0.18871 (17)	0.78713 (16)	0.44180 (11)	0.0432 (3)	
C3	0.22510 (18)	0.79071 (17)	0.53777 (11)	0.0478 (4)	
C4	0.26271 (18)	0.66134 (18)	0.60108 (11)	0.0485 (4)	
C5	0.26484 (17)	0.52696 (17)	0.56774 (10)	0.0430 (3)	
C6	0.23057 (14)	0.51821 (14)	0.47044 (9)	0.0344 (3)	
C7	0.23780 (14)	0.37460 (13)	0.43405 (9)	0.0345 (3)	
C8	0.2708 (2)	0.23544 (17)	0.50650 (11)	0.0475 (3)	
C9	0.22683 (18)	0.24468 (14)	0.29458 (10)	0.0387 (3)	
C10	0.26636 (16)	0.28390 (14)	0.17541 (9)	0.0379 (3)	
C11	0.4468 (2)	0.35385 (19)	0.13855 (13)	0.0535 (4)	
C12	0.1233 (2)	0.38992 (17)	0.13821 (11)	0.0502 (4)	
C13	0.27857 (16)	0.14083 (14)	0.12936 (10)	0.0378 (3)	
C14	0.09534 (15)	−0.05311 (13)	0.12181 (8)	0.0323 (3)	
C15	0.24475 (17)	−0.12727 (17)	0.05479 (11)	0.0411 (3)	
C16	−0.07908 (15)	−0.12069 (13)	0.15177 (8)	0.0329 (3)	
C17	−0.10843 (17)	−0.25178 (15)	0.12019 (10)	0.0395 (3)	
C18	−0.26901 (18)	−0.31863 (17)	0.15062 (11)	0.0464 (3)	
C19	−0.40732 (18)	−0.25416 (18)	0.21474 (10)	0.0474 (3)	
C20	−0.38434 (17)	−0.12466 (17)	0.24715 (10)	0.0452 (3)	
C21	−0.22264 (16)	−0.05565 (15)	0.21614 (9)	0.0384 (3)	
H01	0.170 (2)	0.545 (2)	0.3073 (15)	0.079 (6)*	
H2	0.162 (2)	0.8773 (19)	0.3937 (13)	0.054 (4)*	
H3	0.223 (2)	0.8850 (18)	0.5615 (12)	0.050 (4)*	
H4	0.291 (2)	0.664 (2)	0.6686 (15)	0.072 (5)*	
H5	0.292 (2)	0.4369 (19)	0.6115 (13)	0.054 (4)*	
H8B	0.194 (2)	0.2313 (19)	0.5766 (14)	0.065 (5)*	
H8C	0.388 (3)	0.231 (2)	0.5152 (16)	0.084 (6)*	
H8A	0.246 (3)	0.148 (3)	0.4832 (17)	0.089 (6)*	
H9A	0.327 (2)	0.1723 (18)	0.3147 (12)	0.054 (4)*	
H9B	0.115 (2)	0.1914 (18)	0.3175 (12)	0.047 (4)*	
H11A	0.476 (2)	0.375 (2)	0.0579 (15)	0.068 (5)*	
H11C	0.542 (3)	0.285 (2)	0.1658 (14)	0.072 (5)*	
H11B	0.444 (2)	0.445 (2)	0.1650 (14)	0.065 (5)*	
H12C	0.117 (2)	0.482 (2)	0.1598 (14)	0.066 (5)*	
H12B	0.140 (2)	0.4148 (19)	0.0638 (14)	0.062 (5)*	
H12A	−0.003 (3)	0.344 (2)	0.1627 (15)	0.072 (5)*	
H13B	0.3711 (19)	0.0670 (17)	0.1561 (11)	0.044 (4)*	
H13A	0.3126 (19)	0.1690 (16)	0.0504 (12)	0.044 (4)*	
H02	−0.080 (3)	0.098 (2)	0.2212 (15)	0.078 (6)*	
H20	−0.481 (2)	−0.078 (2)	0.2925 (13)	0.062 (5)*	
H19	−0.523 (2)	−0.3005 (19)	0.2356 (13)	0.061 (4)*	
H18	−0.285 (2)	−0.4125 (19)	0.1276 (12)	0.058 (4)*	
H17	−0.012 (2)	−0.2981 (17)	0.0743 (12)	0.051 (4)*	
H15A	0.353 (3)	−0.080 (2)	0.0387 (16)	0.084 (6)*	
H15B	0.274 (3)	−0.223 (3)	0.0876 (16)	0.082 (6)*	
H15C	0.212 (2)	−0.149 (2)	−0.0053 (15)	0.070 (5)*	

### Atomic displacement parameters (Å^2^)

**Table:**

	*U*^11^	*U*^22^	*U*^33^	*U*^12^	*U*^13^	*U*^23^
O1	0.0605 (6)	0.0362 (5)	0.0397 (5)	−0.0023 (4)	−0.0153 (4)	−0.0034 (4)
O2	0.0479 (5)	0.0556 (6)	0.0603 (6)	0.0028 (5)	0.0009 (5)	−0.0310 (5)
N1	0.0416 (5)	0.0328 (6)	0.0345 (5)	−0.0006 (4)	−0.0063 (4)	−0.0067 (4)
N2	0.0390 (5)	0.0333 (6)	0.0367 (5)	0.0016 (4)	−0.0063 (4)	−0.0098 (4)
C1	0.0323 (5)	0.0390 (7)	0.0343 (6)	−0.0049 (5)	−0.0006 (5)	−0.0070 (5)
C2	0.0430 (6)	0.0372 (7)	0.0460 (7)	−0.0058 (5)	0.0006 (6)	−0.0083 (6)
C3	0.0446 (7)	0.0480 (8)	0.0510 (8)	−0.0114 (6)	0.0043 (6)	−0.0220 (6)
C4	0.0467 (7)	0.0618 (10)	0.0400 (7)	−0.0077 (6)	−0.0038 (6)	−0.0203 (6)
C5	0.0413 (7)	0.0511 (8)	0.0368 (6)	−0.0024 (6)	−0.0072 (6)	−0.0090 (6)
C6	0.0290 (5)	0.0396 (7)	0.0335 (6)	−0.0023 (4)	−0.0018 (5)	−0.0081 (5)
C7	0.0297 (5)	0.0364 (7)	0.0354 (6)	−0.0016 (4)	−0.0035 (5)	−0.0044 (5)
C8	0.0551 (8)	0.0426 (8)	0.0423 (7)	0.0012 (6)	−0.0104 (7)	−0.0013 (6)
C9	0.0471 (7)	0.0303 (6)	0.0381 (6)	−0.0020 (5)	−0.0072 (5)	−0.0062 (5)
C10	0.0452 (6)	0.0306 (6)	0.0368 (6)	−0.0024 (5)	−0.0044 (5)	−0.0074 (5)
C11	0.0588 (9)	0.0448 (9)	0.0543 (8)	−0.0171 (7)	0.0058 (7)	−0.0163 (7)
C12	0.0741 (10)	0.0347 (8)	0.0435 (7)	0.0099 (7)	−0.0168 (7)	−0.0103 (6)
C13	0.0389 (6)	0.0348 (7)	0.0392 (6)	−0.0012 (5)	−0.0036 (5)	−0.0108 (5)
C14	0.0364 (6)	0.0316 (6)	0.0297 (5)	0.0046 (4)	−0.0095 (5)	−0.0061 (4)
C15	0.0378 (6)	0.0407 (8)	0.0460 (7)	0.0037 (5)	−0.0051 (6)	−0.0166 (6)
C16	0.0363 (6)	0.0358 (7)	0.0275 (5)	0.0035 (5)	−0.0099 (5)	−0.0054 (4)
C17	0.0417 (6)	0.0413 (7)	0.0375 (6)	0.0004 (5)	−0.0108 (5)	−0.0099 (5)
C18	0.0488 (7)	0.0499 (8)	0.0445 (7)	−0.0099 (6)	−0.0138 (6)	−0.0099 (6)
C19	0.0398 (7)	0.0662 (10)	0.0367 (6)	−0.0111 (6)	−0.0113 (6)	−0.0023 (6)
C20	0.0370 (6)	0.0622 (9)	0.0351 (6)	0.0033 (6)	−0.0060 (5)	−0.0085 (6)
C21	0.0392 (6)	0.0458 (7)	0.0310 (6)	0.0042 (5)	−0.0084 (5)	−0.0095 (5)

### Geometric parameters (Å, °)

**Table:**

O1—C1	1.3443 (15)	C10—C12	1.527 (2)
O1—H01	1.01 (2)	C10—C11	1.5347 (19)
O2—C21	1.3439 (16)	C10—C13	1.5379 (17)
O2—H02	1.02 (2)	C11—H11A	1.039 (18)
N1—C7	1.2833 (15)	C11—H11C	1.02 (2)
N1—C9	1.4622 (15)	C11—H11B	0.961 (19)
N2—C14	1.2888 (15)	C12—H12C	0.934 (19)
N2—C13	1.4622 (16)	C12—H12B	0.962 (18)
C1—C2	1.3987 (18)	C12—H12A	1.06 (2)
C1—C6	1.4147 (18)	C13—H13B	1.026 (15)
C2—C3	1.380 (2)	C13—H13A	1.022 (15)
C2—H2	0.991 (17)	C14—C16	1.4816 (17)
C3—C4	1.380 (2)	C14—C15	1.5061 (15)
C3—H3	0.973 (16)	C15—H15A	0.94 (2)
C4—C5	1.3844 (19)	C15—H15B	0.94 (2)
C4—H4	0.980 (19)	C15—H15C	0.95 (2)
C5—C6	1.4014 (17)	C16—C17	1.3964 (17)
C5—H5	0.959 (17)	C16—C21	1.4191 (16)
C6—C7	1.4799 (17)	C17—C18	1.3835 (19)
C7—C8	1.4988 (19)	C17—H17	0.984 (15)
C8—H8B	0.994 (18)	C18—C19	1.390 (2)
C8—H8C	0.94 (2)	C18—H18	0.991 (17)
C8—H8A	0.96 (2)	C19—C20	1.374 (2)
C9—C10	1.5374 (17)	C19—H19	0.986 (18)
C9—H9A	1.031 (17)	C20—C21	1.4004 (19)
C9—H9B	0.994 (16)	C20—H20	0.982 (17)
			
C1—O1—H01	101.4 (11)	C10—C11—H11C	110.7 (11)
C21—O2—H02	105.2 (11)	H11A—C11—H11C	110.3 (14)
C7—N1—C9	122.61 (10)	C10—C11—H11B	109.6 (10)
C14—N2—C13	121.02 (9)	H11A—C11—H11B	110.0 (15)
O1—C1—C2	118.27 (12)	H11C—C11—H11B	107.8 (15)
O1—C1—C6	121.67 (11)	C10—C12—H12C	113.3 (11)
C2—C1—C6	120.06 (12)	C10—C12—H12B	113.7 (10)
C3—C2—C1	120.32 (13)	H12C—C12—H12B	103.8 (15)
C3—C2—H2	123.2 (9)	C10—C12—H12A	111.7 (11)
C1—C2—H2	116.5 (9)	H12C—C12—H12A	108.6 (14)
C4—C3—C2	120.55 (13)	H12B—C12—H12A	105.0 (15)
C4—C3—H3	119.5 (9)	N2—C13—C10	112.30 (9)
C2—C3—H3	119.9 (9)	N2—C13—H13B	107.6 (8)
C3—C4—C5	119.67 (13)	C10—C13—H13B	110.9 (8)
C3—C4—H4	120.6 (11)	N2—C13—H13A	108.3 (8)
C5—C4—H4	119.8 (11)	C10—C13—H13A	107.7 (8)
C4—C5—C6	121.73 (14)	H13B—C13—H13A	110.0 (11)
C4—C5—H5	119.8 (10)	N2—C14—C16	117.60 (9)
C6—C5—H5	118.5 (10)	N2—C14—C15	123.32 (11)
C5—C6—C1	117.67 (12)	C16—C14—C15	119.08 (10)
C5—C6—C7	121.47 (12)	C14—C15—H15A	115.5 (12)
C1—C6—C7	120.86 (11)	C14—C15—H15B	112.0 (12)
N1—C7—C6	117.17 (11)	H15A—C15—H15B	102.8 (17)
N1—C7—C8	124.21 (12)	C14—C15—H15C	111.9 (11)
C6—C7—C8	118.62 (11)	H15A—C15—H15C	112.1 (17)
C7—C8—H8B	112.3 (10)	H15B—C15—H15C	101.2 (17)
C7—C8—H8C	109.6 (13)	C17—C16—C21	117.28 (11)
H8B—C8—H8C	107.1 (16)	C17—C16—C14	121.82 (10)
C7—C8—H8A	112.6 (13)	C21—C16—C14	120.88 (11)
H8B—C8—H8A	104.6 (16)	C18—C17—C16	122.33 (12)
H8C—C8—H8A	110.4 (17)	C18—C17—H17	118.7 (9)
N1—C9—C10	110.72 (10)	C16—C17—H17	119.0 (9)
N1—C9—H9A	111.3 (9)	C17—C18—C19	119.50 (13)
C10—C9—H9A	107.0 (8)	C17—C18—H18	120.2 (9)
N1—C9—H9B	111.3 (9)	C19—C18—H18	120.3 (9)
C10—C9—H9B	108.6 (9)	C20—C19—C18	120.05 (13)
H9A—C9—H9B	107.8 (13)	C20—C19—H19	120.2 (10)
C12—C10—C11	110.49 (12)	C18—C19—H19	119.8 (10)
C12—C10—C9	110.72 (10)	C19—C20—C21	120.84 (12)
C11—C10—C9	109.04 (11)	C19—C20—H20	120.5 (10)
C12—C10—C13	110.17 (11)	C21—C20—H20	118.6 (10)
C11—C10—C13	107.19 (10)	O2—C21—C20	118.48 (11)
C9—C10—C13	109.15 (10)	O2—C21—C16	121.53 (11)
C10—C11—H11A	108.6 (10)	C20—C21—C16	119.99 (12)
			
O1—C1—C2—C3	−179.89 (11)	C14—N2—C13—C10	178.35 (10)
C6—C1—C2—C3	−0.12 (18)	C12—C10—C13—N2	−57.39 (14)
C1—C2—C3—C4	−0.5 (2)	C11—C10—C13—N2	−177.66 (11)
C2—C3—C4—C5	0.3 (2)	C9—C10—C13—N2	64.38 (14)
C3—C4—C5—C6	0.4 (2)	C13—N2—C14—C16	−179.70 (10)
C4—C5—C6—C1	−0.91 (18)	C13—N2—C14—C15	0.90 (18)
C4—C5—C6—C7	178.31 (11)	N2—C14—C16—C17	−178.97 (10)
O1—C1—C6—C5	−179.45 (10)	C15—C14—C16—C17	0.45 (17)
C2—C1—C6—C5	0.78 (17)	N2—C14—C16—C21	−0.40 (17)
O1—C1—C6—C7	1.32 (17)	C15—C14—C16—C21	179.02 (11)
C2—C1—C6—C7	−178.45 (10)	C21—C16—C17—C18	−0.89 (19)
C9—N1—C7—C6	177.71 (10)	C14—C16—C17—C18	177.73 (11)
C9—N1—C7—C8	−1.80 (19)	C16—C17—C18—C19	0.1 (2)
C5—C6—C7—N1	−175.41 (11)	C17—C18—C19—C20	0.2 (2)
C1—C6—C7—N1	3.79 (16)	C18—C19—C20—C21	0.2 (2)
C5—C6—C7—C8	4.13 (17)	C19—C20—C21—O2	179.69 (12)
C1—C6—C7—C8	−176.68 (11)	C19—C20—C21—C16	−1.0 (2)
C7—N1—C9—C10	−158.41 (11)	C17—C16—C21—O2	−179.41 (11)
N1—C9—C10—C12	−59.23 (14)	C14—C16—C21—O2	1.96 (18)
N1—C9—C10—C11	62.54 (14)	C17—C16—C21—C20	1.33 (18)
N1—C9—C10—C13	179.33 (10)	C14—C16—C21—C20	−177.30 (11)

### Hydrogen-bond geometry (Å, °)

**Table:**

*D*—H···*A*	*D*—H	H···*A*	*D*···*A*	*D*—H···*A*
O1—H01···N1	1.01 (2)	1.56 (2)	2.517 (1)	156 (2)
O2—H02···N2	1.02 (2)	1.59 (2)	2.526 (1)	151 (2)
C19—H19···O1i	0.99 (2)	2.54 (2)	3.488 (2)	174 (1)
C3—H3···O2^i^	0.97 (2)	2.61 (2)	3.291 (2)	128 (1)

Symmetry codes: i; (i) −*x*, −*y*+1, −*z*+1.
